# Multiple rectal neuroendocrine tumors: An analysis of 15 cases and literature review

**DOI:** 10.3389/fonc.2022.996306

**Published:** 2022-09-14

**Authors:** Shu Pang, Ye Zong, Kun Zhang, Haiying Zhao, Yongjun Wang, Junxiong Wang, Chuntao Liu, Yongdong Wu, Peng Li

**Affiliations:** ^1^ Department of General Practice, Beijing Friendship Hospital, Capital Medical University, Beijing, China; ^2^ Department of Gastroenterology, Beijing Friendship Hospital, Capital Medical University, Beijing, China; ^3^ Department of Pathology, Beijing Friendship Hospital, Capital Medical University, Beijing, China

**Keywords:** rectum, multiple, neuroendocrine tumors, metastasis, diagnosis, treatment, prognosis

## Abstract

Multiple neuroendocrine tumors (M-NETs) are rare in the rectum and there is no consensus on their characteristics and treatments. Here, we report 15 cases of rectal M-NETs and review the previous literature. We discuss the clinical characteristics, endoscopic features and pathological features of rectal M-NETs, aiming to analyze the treatments and follow-up strategies in combination with these characteristics. We retrospectively reviewed and analyzed the data of 15 patients with rectal M-NETs who were diagnosed and treated at Beijing Friendship Hospital, Capital Medical University. Their clinical data, endoscopic findings, pathological features and treatments were analyzed. Follow-up evaluations and literature review were performed. In all, 14 male (93.3%) and 1 female (6.7%) were recruited. The average age at diagnosis was 55.7 years. The clinical manifestations include asymptomatic in 9 patients (60.0%), defecation habits changes in 2 patients (13.3%), anal distension in 2 patients (13.3%), and abdominal distension in 2 patient (13.3%). The largest tumor diameter ≤10mm was found in 13 patients (86.7%) and >10mm in 2 patients (13.3%). All of the lesions originated from the mucous or submucosa layer. WHO grades were all NET G1. The number of tumors diagnosed by pathology in 13 patients was consistent with that observed by endoscopy, while more lesions were observed by pathology than endoscopy in two patients. Lymph node metastasis occurred in 1 patient (6.7%), and vascular or lymphatic invasion occurred in 9 patients (60.0%). Among the 13 patients with the largest tumor diameter being ≤10mm, lymphovascular invasion occurred in 8 patients (61.5%). And among the 2 patients with the largest tumor diameter of >10mm, lymphovascular invasion occurred in 1 patient (50.0%). 14 patients underwent endoscopic resection and 1 underwent surgical excision. Postoperative follow-up was achieved in 13 patients and no recurrence or metastasis was found. The true number of rectal M-NETs may be more than seen under endoscopy. Rectal M-NETs is associated with a high risk of metastasis; therefore, treatment and surveillance strategies should be more radical than single lesion.

## Introduction

The incidence of neuroendocrine neoplasm (NEN) is increasing in the world. Gastrointestinal and pancreatic neuroendocrine neoplasm (GEP-NEN) is the most common. Rectal NENs (r-NENs) represent 12%–27% of all gastrointestinal NENs and 1%–2% of all rectal tumors are neuroendocrine (1). According to the degree of differentiation, NEN can be divided into well differentiated neuroendocrine tumors (NETs) and poorly differentiated neuroendocrine carcinomas (NECs) (2). Most of rectal NETs are single; multiple neuroendocrine tumors (M-NETs) are rare. It is reported that the incidence of rectal M-NETs is between 2% and 4.5% (3).

Rectal NETs are usually accidentally found in screening endoscopies and only a few patients have symptoms. Common symptoms include changes in defecation habits, hematochezia and abdominal pain, but these symptoms may have nothing to do with tumors (4). There are no specific physical exams and lab results for M-NETs. The choice of treatment for rectal NETs depends on the size of the tumors, the depth of invasion and the presence of distant metastasis (3). However, due to the rarity of rectal M-NETs, most of the literature related to M-NETs in the past appeared in the form of case reports. There is no consensus on their characteristics and treatments. Therefore, it is necessary to accumulate similar cases. In this study, we will report 15 cases of rectal M-NETs in our hospital and review the previous literature, aiming to discuss treatments and follow-up strategies in combination with clinical, endoscopic and pathological features.

## Materials and methods

This study was approved by the ethics committees of Beijing Friendship Hospital, Capital Medical University. We retrospectively reviewed and analyzed the data of patients with rectal M-NETs found under endoscopy and confirmed by pathology and treated in Beijing Friendship Hospital, Capital Medical University between November 2012 and May 2022. A total of 15 patients were included in this study. We collected clinical data of the cases, including gender, age at diagnosis, clinical symptoms, tumor marker test results, endoscopic features, pathological and immunohistochemistry results and treatments. The number, location, size and invasion depth of the lesions were evaluated by professional endoscopists under electronic colonoscopy and endoscopic ultrasonography. Biopsy or surgical specimens were evaluated by pathologists and immunohistochemical staining and special staining were performed to confirm the diagnosis and evaluate lymphovascular invasion and lymph node metastasis.

Patients were advised to follow up in the outpatient department after operation and the follow-up period depends on the characteristics of the lesions. Recurrence and metastasis were monitored by abdominal and pelvic enhanced CT or electronic colonoscopy. The follow-up results were based on the latest outpatient medical record or electronic examination report, and the patients without postoperative reexamination records were followed up by telephone to find out the specific situation. The follow-up time was from surgical treatment to the latest outpatient visit.

We reviewed the literature related to rectal M-NETs in PubMed up to May 2022. The search command used in the search was (neuroendocrine tumors) and (multiple) and (rectal or rectum). Cases with complete clinical data and pathologically confirmed as M-NETs were included. When there was no full text of the article, or the clinical data of the cases reported in the article was incomplete or the patient were not pathologically diagnosed as M-NETs, it was excluded.

## Results

### Patient demographics and clinical characteristics

Detailed demographics and clinical characteristics are summarized in **Table 1**. Of the fifteen cases, 93.3% (14/15) of the patients were male. The age at diagnosis ranged from 44 to 80 years, with an average age of 55.7 years. Among them, 9 cases (60.0%) were asymptomatic and lesions were accidentally found during routine physical examination, 2 cases (13.3%) (case 3 and 15) showed changes in defecation habits, 2 cases (13.3%) (case 2 and 10) showed a feeling of anal distension, and the other 2 cases (13.3%) (case 11 and case14) showed abdominal distension. None of the 15 patients had a family history of neuroendocrine tumors or digestive tract tumors. One case (case12) had undergone partial hepatectomy and orthotopic liver transplantation for hepatocellular carcinoma.

**Table 1:** Summary of our fifteen cases.

Case	Year	Sex	Age	Symptom	Tumor markers	Number	Size (mm)	The depth of invasion	Location	Grading	Lymphatic invasion	Venous invasion	Lymph node metastasis	Distant metastasis	Treatment	Follow-up	Recurrence
1	2021	Male	50	No	Normal	2	2,10	Submucosa	10cm from the AV	NET G1	No	Yes	No	No	ESD	No	Unknown
2	2021	Male	62	Anal Heaviness	Normal	2	5, 14	Mucosa	5,4cm from the AV	NET G1	No	No	No	No	ESD	11	No
3	2020	Male	60	Changes in Defecation Habits	Normal	3	4	Submucosa	Rectum	NET G1	No	No	No	No	ESD	19	No
4	2019	Male	54	No	Normal	2	4	Submucosa	8cm from the AV	NET G1	No	No	No	No	ESD	16	No
5	2019	Female	48	No	CA125↑	2	3,5	Submucosa	12,8cm from the AV	NET G1	No	No	No	No	ESD	24	No
6	2019	Male	66	No	Normal	3	5,5,8	Submucosa	10,8,5cm from the AV	NET G1	Yes	Yes	No	No	ESD	30	No
7	2021	Male	44	No	Normal	3	6,4,3	Submucosa	11,10,8cm from the AV	NET G1	Yes	Yes	No	No	ESD	7	No
8	2012	Male	47	No	Normal	2	3	Submucosa	Rectum	NET G1	No	No	No	No	EMR	Lost	Unknown
9	2021	Male	61	No	Normal	3	6,6,8	Submucosa	12,12,10cm from the AV	NET G1	Yes	No	No	No	ESD	No	Unknown
10	2016	Male	80	Anal Heaviness	CA199↑	2	4,7	Submucosa	8cm from the AV	NET G1	No	Yes	No	No	ESD	No	Unknown
11	2022	Male	45	Abdominal Distension	Normal	7	2-6	Submucosa	3-10cm from the AV	NET G1	Yes	No	No	No	ESD	No	Unknown
12	2018	Male	46	No	AFP↑	2	2,6	Submucosa	7,2cm from the AV	NET G1	No	No	No	No	ESE	Lost	Unknown
13	2021	Male	52	No	CA724↑	6	4	Submucosa	Rectum	NET G1	Yes	Yes	No	No	ESD	7	Unknown
14	2021	Male	63	Abdominal Distension	Normal	8	3-12	Submucosa	Rectum	NET G1	Yes	Yes	No	No	EMR+ESD	No	Unknown
15	2021	Male	57	Changes in Defecation Habits	Normal	6	4-30	Submucosa	Rectum and Sigmoid Colon	NET G1	Yes	Yes	Yes	No	TME	10	No

AV, anal verge; ESD, endoscopic submucosal dissection; EMR, endoscopic mucosal resection; ESE, endoscopic submucosal excavation; TME, total mesorectal excision.

### Laboratory and imaging features

Serum tumor markers were examined in all 15 patients, including 11cases in the normal range, 1 case of elevated CA125 (case5), 1 case of elevated CA199 (case10), 1 case of elevated AFP (case12), and 1 case of elevated CA724 (case13). All patients underwent abdominal-pelvic enhanced CT or abdominal MRI, where no pancreatic or upper gastrointestinal lesions were found, and no lymph node or distant metastasis were found.

### Endoscopic findings

**Figure 1:**
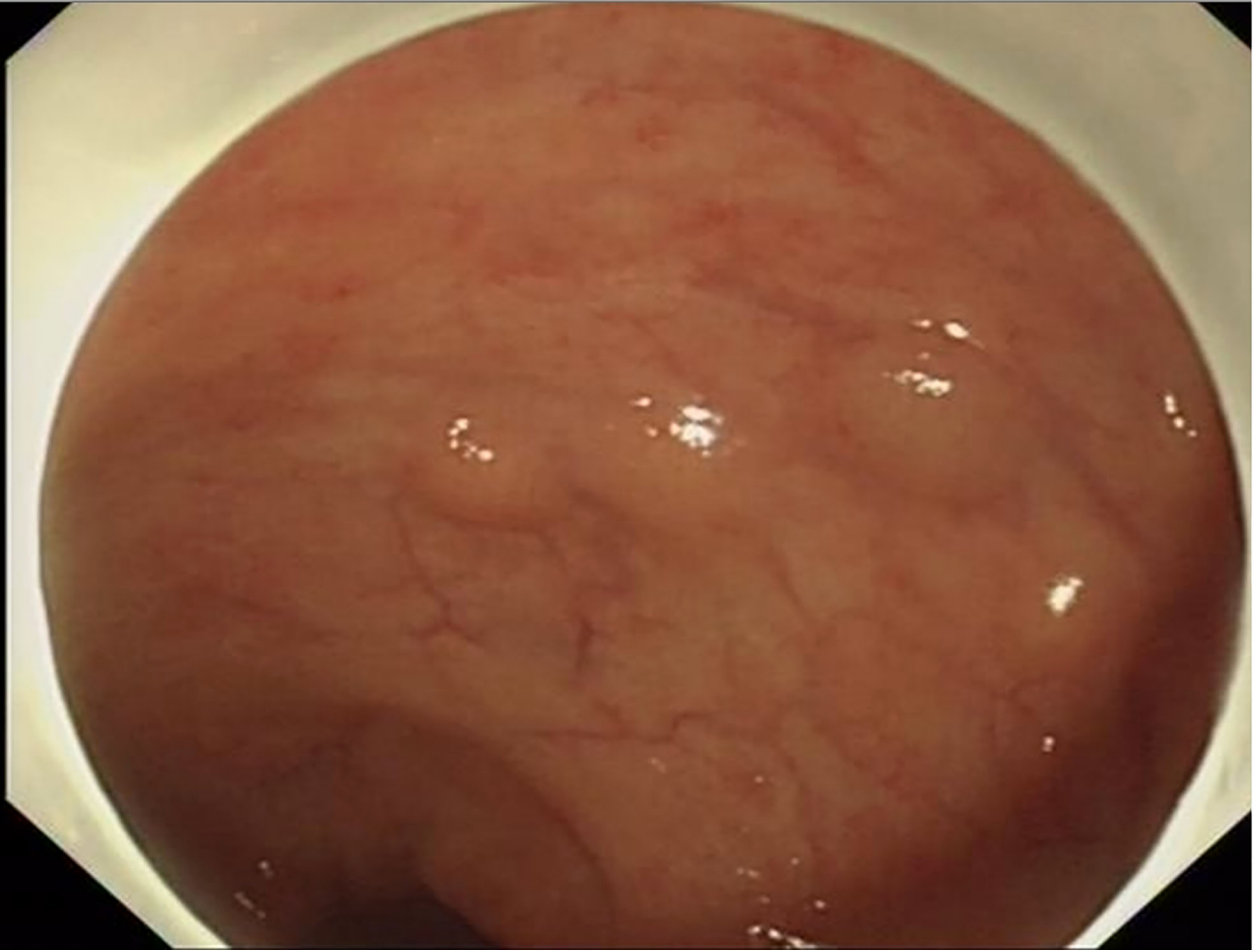
Most NET lesions showed as yellow or white bulge under endoscopy, and the surface mucosa was smooth.

**Figure 2:**
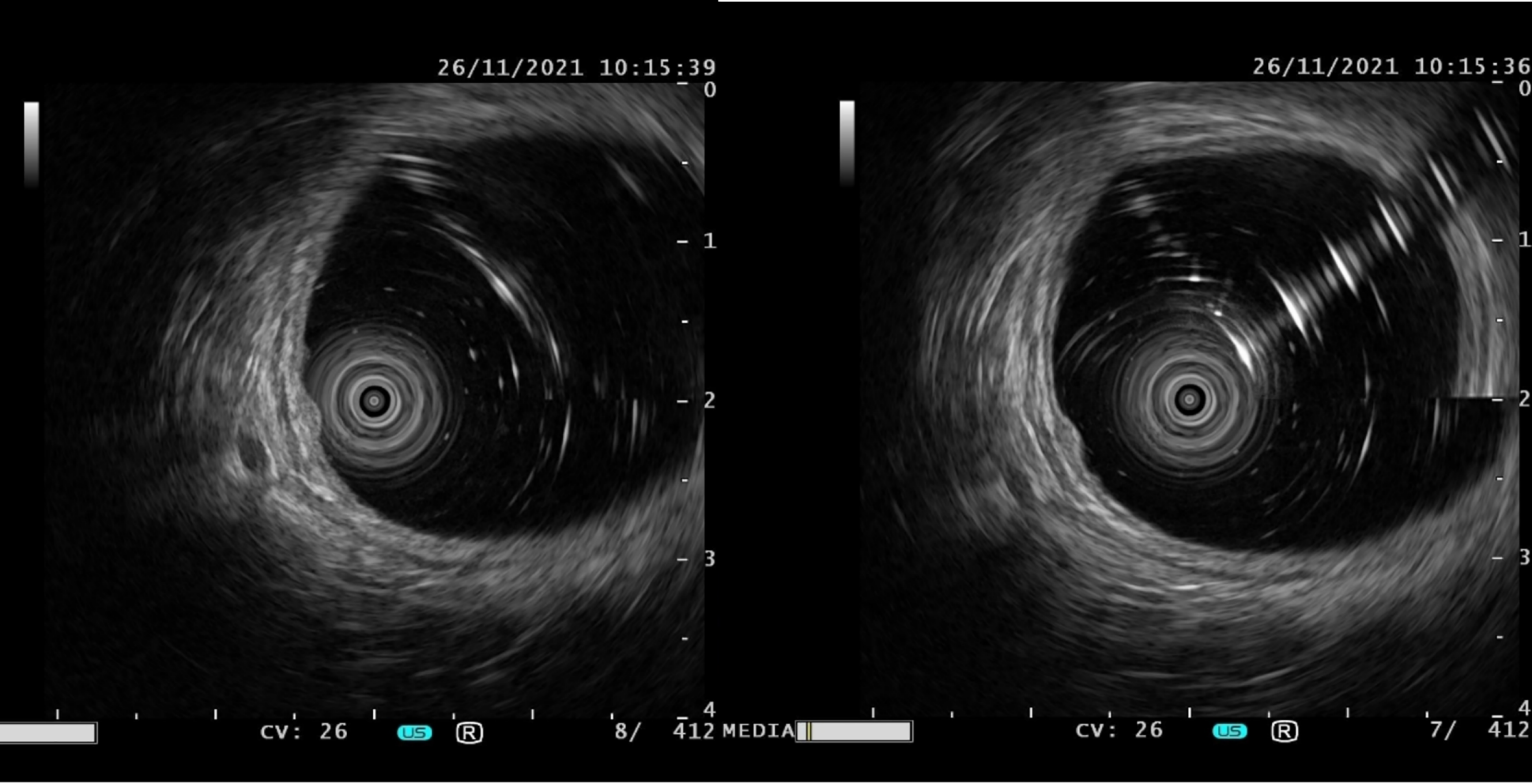
The typical manifestations of lesions under endoscopic ultrasonography were originated from the mucous layer or submucosa, and the internal echo was homogeneous, showing low or moderately low echo, and the boundary was clear.

Among the 15 patients, only case15 had lesions in both the rectum and sigmoid colon, and the remaining 14 patients had lesions confined to the rectum. The location of the lesions ranged from 2 to 12 cm from the anal verge, with a median distance of 8 cm. The number of lesions under endoscopy ranged from 2 to 8, with a median number of 3. The size of lesions ranged from 2 to 14mm. The largest tumor diameter ≤10mm was found in 13 cases (86.7%), except that the maximum diameter of case 2 was 14mm and case 14 was 12mm. Most lesions showed as a yellow or white bulge under endoscopy, and the surface mucosa was smooth (**Figure 1**). Uniquely in case 15, one adenocarcinoma lesion and 5 small smooth polyps with yellow and smooth surface mucosa were scattered in the rectum and sigmoid colon. Endoscopic ultrasonography was performed in case1, 2, 4, 6, 7, 9, 10, 12, 13 and 14, and the results showed that all of the lesions originated from the mucous layer or submucosa, and the internal echo was homogeneous, showing low or moderately low echo, and the boundary was clear (**Figure 2**).

### Pathological, immunohistochemical and special staining findings

The NETs in all patients were limited to the mucosa and submucosa, and the postoperative pathological WHO grades were all NET G1. Uniquely, the adenocarcinoma lesion of case 15 was consisted of moderately differentiated adenocarcinoma and NET, with a clear boundary between the two components. The number of tumors found by pathology was not completely consistent with that seen by endoscopy. Specifically, case 11 showed 7 bulges under endoscopy, and an additional small lesion was observed by pathology. There were 6 lesions scattered in the rectum of case 15, and more lesions were found in the postoperative pathological examination. Only case 15 showed lymph node metastasis and neuroendocrine tumors (G1) were found in 11 of 31 periintestinal lymph nodes. The rate of lymph node metastasis was 6.7% (1/15). Among the 15 patients, lymphovascular invasion occurred in 9 patients (60.0%), including vascular invasion in 7 patients (46.7%) and lymphatic invasion in 7 patients (46.7%). Among the 13 patients with the largest tumor diameter ≤10mm, lymphovascular invasion occurred in 8 patients (61.5%). And among the 2 patients with the largest tumor diameter >10mm, lymphovascular invasion occurred in 1 patient (50.0%).

### Treatment and follow-up

Eleven patients were treated by endoscopic submucosal dissection (ESD), one patient was treated by endoscopic mucosal resection (EMR) (case 8), one patient was treated by endoscopic submucosal excavation (ESE) (case 12), one patient was treated by EMR and ESD (case 14), and one patient was treated by total mesorectal excision (TME) and transanal endoscopic microsurgery (TEM) (case 15).

Two patients lost follow-up (case 8 and 12). Postoperative follow-up was achieved in the rest thirteen patients. Seven patients were re-examined by abdominal-pelvic enhanced CT or electronic colonoscopy regularly, and no recurrence or metastasis was found. Five patients (case 1, 9, 10, 11 and 14) did not undergo any re-examination after treatment, some because the re-examination time was not reached, and some were unable to see a doctor because of the epidemic of novel coronavirus. One patient (case 13) underwent partial enterectomy in other hospital five months after our treatment and the re-examination time has not come until the deadline of this article.

## Discussion

With the popularity of colonoscopy screening, the detection rate of early rectal NET has increased significantly in recent years. 80-90% of rectal NET are diagnosed incidentally by colonoscopy (5). Rectal NET usually occurs singly, while multiple tumors are rare. Although progress has been made in the study of rectal NEN, many aspects of the disease are still unclear, in part due to its rarity. To study the clinical characteristics of rectal M-NETs, we reviewed the previous case reports and found nineteen articles reporting a total of 27 cases with relatively complete clinical data. The data of the patients in the literature are shown in **Table 2**. The number of M-NETs in these cases ranged from 2 to 69, except that some cases only describe “numerous”. Most patients were treated with local resection. Among them, 2 cases complicated with UC, 6 cases complicated with other gastrointestinal malignancies.

**Table 2:** Summary of multiple rectal carcinoid case reports.

Literature	Country and References	Symptom	Carcinoid syndrome	Tumor markers	Family history of malignant tumors	Sex	Age(years)	Lymphaticinvasion	VenousInvasion	Lymph nodemetastasis	Distant metastasis	Treatmentmethods	Follow-up(months)	Recurrence	Number under Endoscope	Number under Microscope	Size(mm)	Shape	Location	Depth	WHO grade	Others
(5)	Korea	No	No	Normal	ND	Male	52	ND	ND	ND	No	ESMR-L	18	No	3	3	≤4	SEL	Rectum	Submucosa	NET G1	
	Korea	No	No	Normal	No	Male	32	ND	ND	ND	No	ESMR-L	18	No	3	3	5,5,7	SEL	Rectum	Submucosa	NET G1	
	Korea	No	No	CEA↑	ND	Female	65	ND	ND	ND	No	EMR	Lost	Unknown	3	3	5,6,7	Sessile Polyp	8,10 and 11 cm from the AV	Mucosa	Unknown	
	Korea	No	No	ND	ND	Male	62	No	No	No	No	ESMR-L	12	No	2	2	5,5	SEL	Rectum	Submucosa	NET G1	Complicated with Stomach Cancer
	Korea	No	ND	ND	Yes	Female	48	No	No	No	No	ESMR-L	24	No	2	2	ND	SEL	Rectum and sigmoid colon	Submucosa	NET G1	
(6)	Brussels	Abdominal Pain	No	ND	No	Male	54	ND	ND	ND	No	Partial rectal resection	ND	Unknown	0	10	1-6	No	Rectum	ND	NET G2	Complicated with UC
(7)	Japan	Positive Occult Blood	No	ND	ND	Male	51	No	ND	Yes	No	EMR	120	No	2	69	≤8	SEL	2-12 cm above the AV	Submucosa	NET G1	
	Japan	Positive Occult Blood	No	ND	ND	Male	58	No	ND	Yes	No	Partial Rectal Resection	12	No	31	62	≤7	SEL	Whole Rectum	Submucosa	NET G2	
(8)	China	Bloody stool	No	Normal	No	Female	39	ND	ND	Suspicious	Suspicious	Follow-up every 3-6 Months	ND	Unknown	Multiple	> 30	3-25	SEL	1-10 cm from the AV	Submucosa	NET G1	Neurofibromatosis Type 1-associated
(9)	America	No	ND	ND	ND	Female	53	ND	ND	ND	No	Follow-up every 12 Months	12	Unknown	2	6	2-3	SEL	Distal Rectum	Submucosa	ND	Neurofibromatosis Type 1-associated
(10)	America	Abdominal Pain, Diarrhea	Yes	Normal	ND	Male	52	ND	ND	ND	No	Surgical Operation	9	No	1	3	10-30	Sessile Polyp	2-5cm from the AV	Submucosa	ND	
(11)	Japan	Bloody stool	ND	ND	ND	Male	52	ND	ND	ND	No	AR	ND	No	5	41	4,5,6,9,10	SEL	Rectum	4submucosa 1subserosa	ND	Complicated with Colon Adenocarcinoma
(12)	America	Shortness of Breath	ND	ND	ND	Male	60	ND	ND	ND	ND	No	0	die	Innumerable		≤10	SEL	Rectum	Lamina propria	ND	Died of a Ruptured Dissecting Aneurysm
(13)	Japan	Abdominal Discomfort	No	Normal	ND	Male	64	Yes	Yes	Yes	No	LAR + subTME	156	No	4	15	2-9	ND	Rectum	Submucosa	NET G1	
	Japan	Diarrhea	No	Normal	ND	Male	63	No	No	Yes	No	LAR + subTME	240	No	3	9	2-9	ND	Rectum	Submucosa	NET G1	
	Japan	Positive Occult Blood	No	Normal	ND	Male	50	No	No	No	No	LAR + subTME	120	No	2	2	5, 10	ND	Rectum	Submucosa	NET G1	
(14)	Japan	Recurrent Alternating Constipation and Diarrhea	No	Normal	yes	Male	61	No	No	No	No	ISR	61	No	8	42	<1-6	SEL	Lower rectum	Submucosa	NET G1	
	Japan	No	No	Normal	yes	Male	61	No	No	No	No	ISR	58	No	13	36	<1-5	SEL	Rectum	Submucosa	NET G1	
(15)	Japan	No	ND	Normal	No	Male	69	No	No	No	No	APR	6	No	100	30	<10	SEL	Lower rectum	Submucosa	ND	Complicated with Ganglioneuromas
(3)	Korea	No	No	ND	ND	Male	57	No	No	Yes	No	EMR+TATME	4	No	Multiple	42	≤5	SEL	10 cm from the AV	Submucosa	NET G1	
(16)	China	Bloody Stool	No	ND	ND	Male	47	No	No	No	No	TEM	6	No	3	3	5-8	Sessile Polyp	6 to 8 cm above the AV	Submucosa	NET G1	
(17)	America	No	No	Normal	ND	Female	57	No	No	No	No	Removed via Sigmoidoscope	ND	Unknown	2	2	8,6	SEL	6cm from the AV, Terminal Ileum	ND	ND	
(18)	British	Bloody Stool	Yes	ND	ND	Male	50	ND	ND	ND	No	Total Colectomy with Anal Excision	12	No	0	16	≤2	ND	Distal left Colon	ND	ND	Complicated with UC, Cecum Adenocarcinoma
(19)	China	Bloody Stool	No	ND	ND	Male	70	ND	ND	ND	ND	Dixon	ND	Unknown	2	2	15, 25	SEL	10 cm from the AV	Submucosa	ND	Complicated with Rectum Adenocarcinoma
(20)	Japan	Abdominal Discomfort	No	ND	ND	Male	54	No	No	No	No	EMR	ND	Unknown	4	4	≤6	SEL	Rectum	Submucosa	ND	
(21)	America	Bloody Stool	No	Normal	ND	Male	50	ND	ND	Yes	No	LAR	6	No	3	17	≤10	SEL	Rectum	Submucosa	ND	Complicated with Colon Villous Adenoma

APR, Abdominoperineal resection; AR, Anterior resection; ESMR-L, Endoscopic submucosal resection with a ligation device; EMR, Endoscopic mucosal resection; TEM, Transmission electron microscope; SM: Submucosa; M3: Mina muscularismiucosae; UC, ulcerative colitis

### General and clinical characteristics

Only a small number of patients have carcinoid syndrome, and often are related to peptides and hormones secreted from the primary site. Most M-NETs patients have no typical symptoms, it is difficult to predict accurate prevalence (8). Only one case (18) was clearly reported with carcinoid syndrome. Rectal NET can occur at any age, but it is more common in middle-aged and elderly patients. The average age at diagnosis was 58.2 years old, which is about 10 years younger than other types of tumors (22). In previous case reports of M-NETs, the age of onset ranged from 32 to 70 years, and the average age was 55.0 years, which was almost consistent with the 15 patients we reported (55.7 years), and was close to the median age of onset of NET. The male-to-female ratio of rectal NET is about 1.06, and the African American to white ratio is about 0.34 (22). But these ratios in M-NETs have not been reported by large sample experiment. The male-to-female ratio in the previous reports we reviewed was 22:5, and the ratio of our study was 14:1, which is quite different from the male-to-female ratio of single NET. Perhaps we can boldly speculate that men are more likely to develop rectal M-NETs. A case-control study had reported that family history of cancer was an important risk factor for NETs, and that genetic factors may contribute the most to their occurrence (23). However, only one article (14) reported M-NETs in monozygotic twins, and none of our patients had a family history of cancer. This may be attributed to the small sample size of our study.

### Tumor markers

Rectal M-NETs have not been reported to be associated with specific tumor markers. A recent study collected six serum tumor markers, including alpha-fetoprotein (AFP), carcinoembryonic antigen (CEA), cancer antigen 19-9 (CA19-9), cancer antigen 72-4 (CA72-4), cytokeratin 19 fragment 21-1 (Cyfra21-1) and neuron-specific enolase (NSE), and compared the distribution of all these serum tumor markers in the study participants. Among the six serum tumor markers, only NSE was significantly associated with the histologic grades in GEP-NETs (24). Only one case (5) reported the increase of CEA. Including our 15 patients, there was no increase in NSE, which may be related to the small sample size. The relationship between tumor markers and M-NETs remains to be supported by more data.

### Endoscopic characteristics

Rectal M-NETs have unique appearance under endoscopy, but there is also the possibility of misdiagnosis and missed diagnosis. Typically, NETs appear as smooth, round submucosal nodules with yellow or white mucosa under endoscope. A few present as sessile polyps (25). Rectal NETs are often located in the mid-rectum (4 to 8 cm from the anorectal junction) (26), which is consistent with our data of M-NETs. Therefore, if a lesion is observed at this location, the endoscopist needs to be alert to whether the lesion is a neuroendocrine tumor and to carefully observe whether there are more lesions. About 80% of NETs are 10 mm or less in size and are contained within the submucosa at the time of diagnosis (27). Multiple biopsies had been reported to be the initial diagnosis of M-NETs. Additionally, rectal M-NETs may have more lesions that cannot be recognized by endoscope. In 12 previously reported cases (**Table 2**), the number of lesions under endoscopy was less than the actual number; this is largely because lesions were in the initial or intermediate stages of carcinoid tumor development (defined as micronests) with small size and almost normal mucosa and difficult to find under endoscopy (7). The compression of gas or water injection during colonoscopy procedure may also be one of the reasons for the differences. In our report, case 11 and case 15 were also observed more lesions by pathology. When rectal NET is found by accident, the possibility of multiple lesions should be considered, and other sites should be carefully examined.

### Risk factors of lymph node metastasis

Initial diagnosis and assessment of risk factors of metastasis might be important consideration in rectal NETs, as early detection and prompt prediction of metastasis can help patients prolong their survival time. Risk factors associated with lymph node metastasis of rectal NETs mentioned in previous studies mainly include number of lesions, size of the tumor (larger than 10 mm), invasion depth, and the lymphovascular invasion. In 1987, Kanter (21) first proposed that the number of rectal NETs is related to lymphatic metastasis. Patients with multiple tumors are at a high risk for lymph node metastasis regardless of tumor size. Compared with isolated tumors less than 1 cm in size, multiple rectal carcinoid tumors measuring less than 1 cm have a higher incidence of lymph node metastasis (10% to 22.7%) (28). These conclusions were later confirmed in a single-center retrospective study (29). Kasuga (30) et al. found that lymph node metastasis occurred in 11.7% of cases (limited to the mucosa or submucosa) and 87.5% of cases (into or through the muscularis propria). The likelihood of lymph node metastasis increases with tumor size. In tumors smaller than 10mm, lymph node metastasis is a rare result, with an incidence of 1% ~ 10% (31–33). The rectal NEN with a maximum diameter of 1-2 cm has a lymph node metastasis rate of about 30% at the time of diagnosis, and about 40% in those ≥20 mm (27, 34). In the latest multicenter retrospective study, diameter > 11.5 mm and vascular infiltration were independently correlated with nodal involvement (35). It is reported that 22.7% of patients with multiple rectal NETs whose diameter is less than 10mm have lymphatic metastasis (36). Even in tumors 5 to 9 mm in size, 8.4% of metastases were reported (31). As a result, even if the rectal NETs are small, CT, MRI, positron emission tomography, and endoscopic ultrasonography may be necessary to evaluate lymph nodes and distant metastases. In a multivariate analysis of patients with small rectal NETs (≤10 mm), only venous invasion was independently associated with metastasis (30).

In our reported patients and previously reported cases, no distant metastases occurred. Lymph node metastasis occurred in 6 patients in the past reports (**Table 2**) and 1 patient in our study. The tumor sizes of these 7 cases were all ≤10mm and were limited to the submucosa. Among the 7 patients, only 2 cases had lymphovascular invasion, although the incidence of lymphovascular invasion was high in patients with rectal M-NETs. And the number of lesions ranged from 9 to 69. This result supports the conclusion that the number of rectal NET is related to Lymph node metastasis, regardless of the size of the lesions.

### Treatment strategies

Since M-NETs is associated with a high risk of lymph node metastasis, monitoring and treatment strategies should be different from those of single rectal NETs (3). For single rectal NET, small lesions (≤10 mm) without muscularis propria invasion and no metastases found on imaging, complete resection with negative margins under endoscopy is the first choice for treatment (37). However, treatment for multiple rectal neuroendocrine tumors measuring ≤10 mm is not clear. Multiple rectal neuroendocrine tumors even if they are ≤10 mm in size without muscularis propria invasion may have malignant potential and can be effectively treated *via* radical resection (13). For rectal NETs larger than 20 mm, surgical resection is recommended due to the high metastatic risk and involvement of muscularis propria (34). The metastasis risk of rectal NETs of 10-19mm is reported to be 10-15%, and the treatment is controversial (26). The tumor should be fully evaluated by endoscopy and imaging examination, and then appropriate treatment can be discussed. Patients with lymphatic or venous invasion are at high risk of lymph node metastasis, so surgery resection with lymph node dissection should be performed as a radical therapy no matter the size and number of tumors (29).

There are various surgical methods, including EMR, ESD, TEM, anterior resection (AR), abdominoperineal resection (APR) and so on. EMR and ESD are considered to be good options for small NETs. Most NETs occur deep in the epithelial glands and form nodular lesions in the submucosa after penetrating the muscularis mucosa. Therefore, conventional endoscopic polypectomy (EMR and ESD) may be incomplete (16). In contrast, TEM enables wider resection and ensures better tumor outcomes for lesions with malignant potential (16). Among the 6 previously reported patients with lymph node metastasis (**Table 2**), only one patient (7) was treated with EMR, and the rest were treated with surgical radical resection. All patients with lesions >10mm, with lymphatic or venous invasion underwent surgical radical resection. In our study, 1 patient underwent radical enterectomy because of adenocarcinoma, 1 patient underwent enterectomy because of lymphovascular invasion and 13 patients underwent endoscopic resection. Among the 13 patients underwent endoscopic resection, lymphatic or venous invasion occurred in 7 patients, and endoscopic resection may be not enough for them. Fortunately, none of them relapsed until the last re-examination (some patients did not arrive for the re-examination time). As the follow-up time increases, we may prove that radical treatment may not be necessary.

### Prognosis and surveillance

In order to reduce the psychological burden of patients and improve the overall quality of life, surveillance protocol is necessary. The diameter of the primary neoplasm, the number of positive nodes (≥ 5 as a cut-off) and the depth of invasion with the invasion of the muscolaris propria are considered to be the main independent predictors of metastatic spread and dismal prognosis (1). Patients with small (≤1 cm) and confined to the submucosa rectal NET have a 5-year survival of 98–100% and post-treatment monitoring is not recommended currently. While those with regional and distant metastases have a survival of 54–74% and 15–37% respectively. If a tumor has distant metastasis or regional lymph node metastasis, invades peritoneum or other organs, invades muscularis propria or size more than 2 cm should have 6-monthly surveillance for 5-10 years with CT of the chest, abdomen and pelvis. For rectal NETs 1–2cm in size, colonoscopy, EUS and MRI at 12 months is necessary (27). There is no statistical report on the survival rate and no surveillance protocol of M-NETs. However, it should be noted that in the case of M-NETs, relatively short-term endoscopy may be needed to avoid missing other residual NET lesions, and for highly invasive tumors, long-term rather than frequent monitoring is emphasized. In the recorded reports, the follow-up period ranged from 0 to 240 months, with a median follow-up time of 12 months. And all patients survived except 1 patient who died of a ruptured dissecting aneurysm. In our patients, 7 patients underwent regular postoperative re-examination. Our median follow-up time was 13.5 months, and no recurrence was found in all patients. 4 of the 9 patients with lymph node metastasis or lymphovascular invasion were followed up regularly after surgery, with an average follow-up time of 13.5 months. No metastasis or recurrence occurred. Rectal NET is a tumor with relatively slow growth. One of the disadvantages of our study is that the follow-up time is short and the reference value for evaluating patient survival rate is limited.

### Multiple rectal neuroendocrine tumors co-occur with other disease

Rectal M-NETs can co-occur with other malignant tumors. Multiple primary malignant tumors (MPMN) refers to two or more kinds of malignant tumors in an individual, and there is no relationship between these tumors (38). In recent years, the number of reported cases has been increasing. However, there are relatively rare reports of rectal carcinoid and other malignant tumors. In fact, it is reported that up to 55% of patients with gastrointestinal NETs suffer from second primary malignant tumor (39). 6 patients in the articles we reviewed and our case 11 and 15 suffered from MPMN. Treatment is primarily focused on the tumor type that is more histologically aggressive or has predominant behavioural features.

Inflammatory bowel disease (IBD) may promote the development of NETs. Patients with IBD have increased risk of colorectal adenocarcinoma (40). In contrast, colorectal NETs are rarely reported in the context of IBD. Previous reports have questioned whether IBD and NET are related because the incidence of NETs in IBD patients is considered to be similar to that in non-IBD patients, and tumors mainly occur in parts of the intestine not affected by IBD (41). However, recent literature suggests that NETs may be more common in IBD patients than previously thought, and they may be responsive phenomena in IBD to various factors (42). It remains unclear how and to what extent inflammation promotes the development of NETs. In the literature we reviewed, there were two cases complicated with UC. But none of the 15 patients in our study had UC.

## Conclusions

In summary, the true number of rectal M-NETs may be more than seen under endoscopy, mainly because some lesions were in the initial stage of carcinoid tumor development, small and similar to normal mucosa, so it’s difficult to find under endoscopy. Since M-NETs is associated with a high risk of lymph node metastasis, treatment and surveillance strategies should be more radical.

## Data availability statement

The original contributions presented in the study are included in the article/supplementary material. Further inquiries can be directed to the corresponding authors.

## Ethics statement

The studies involving human participants were reviewed and approved by the ethics committees of Beijing Friendship Hospital, Capital Medical University. Written informed consent for participation was not required for this study in accordance with the national legislation and the institutional requirements.

## Author contributions

SP, YZ, YWu and PL were responsible for the conception and design of the study. SP made data collection, data analysis and interpretation, and drafted the manuscript. YZ participated in the idea, protocol design, made English grammar corrections and critically revised important intellectual content. YWu and PL critically revised the important intellectual content and finally approved the version to be released. KZ reevaluated the pathological results. YZ, HZ, YWa, JW and CL were the endoscopists that performed the surgery and did data collection, data analysis, and interpretation. All authors agree to be accountable for the content of the work. All authors contributed to the article and approved the submitted version.

## Funding

This work was supported by the Digestive Medical Coordinated Development Center of Beijing Hospitals Authority No. XXT08. The funder had no role in study design, data collection, analysis and interpretation, decision to submit the article for publication, or preparation of the manuscript.

## Conflict of interest

The authors declare that the research was conducted in the absence of any commercial or financial relationships that could be construed as a potential conflict of interest.

## Publisher’s note

All claims expressed in this article are solely those of the authors and do not necessarily represent those of their affiliated organizations, or those of the publisher, the editors and the reviewers. Any product that may be evaluated in this article, or claim that may be made by its manufacturer, is not guaranteed or endorsed by the publisher.
